# Control of Dental Plaque and Gingival Inflammation by Natural Ingredients-Based Mouthwash

**DOI:** 10.3390/dj14010002

**Published:** 2025-12-19

**Authors:** Mélody Dutot, Marie Le Besco, Océane Mathurin, Shao-Bing Fong, Vincent Meuric, Caroline Tanter

**Affiliations:** 1 YSLAB, 2 rue Félix Le Dantec, 29000 Quimper, France; 2 INSERM, INRAE, UMR 1317, Nutrition, Metabolisms and Cancer (NuMeCan) Institute, Université de Rennes, 2 rue Henri le Guilloux, 35000 Rennes, France; 3 CHU Pontchaillou, Université de Rennes, 2 rue Henri le Guilloux, 35000 Rennes, France

**Keywords:** marine algae, seawater, mouthwash, gingivitis, oral microbiota, dental plaque, natural ingredients, clinical trial

## Abstract

**Background/Objectives**: This study aimed to investigate the effects of a new alcohol-free marine mouthwash containing an algae extract, a coastal plant extract, and seawater on plaque reduction, gingivitis, and oral microbiota balance. **Methods**: In a single-center, prospective, randomized comparative study, 50 subjects with gingivitis were assigned to either a marine mouthwash group (Test, *n* = 26) or a marketed mouthwash group (Comparator, *n* = 24). Clinical assessments included plaque, gingivitis, halitosis, and volunteer self-evaluation at baseline (T0) and after 7 days (T7). Gingival microbiota was sampled using swabs at baseline (T0) and after 7 days (T7). **Conclusions**: Both groups demonstrated reductions in dental plaque, gingivitis, and halitosis at T7 compared to baseline. Improvements in halitosis were observed in both groups but did not reach statistical significance. Microbiota analysis revealed that the Test group experienced the enrichment of health-associated bacterial taxa and a reduction in disease-associated species, notably *Porphyromonas endodontalis*, while the Comparator group showed an increase in pathogenic taxa. The marine mouthwash was well tolerated and positively rated by participants. Combining an algae extract, a coastal plant extract, and seawater, it effectively reduces plaque and gingivitis and may contribute to oral microbiota balance. It represents a promising alternative to conventional chemical oral care products.

## 1. Introduction

Dental plaque represents one of the most complex microbial communities in the human body. Far from being a simple deposit, it is a dynamic biofilm that evolves through the sequential colonization of diverse bacterial species. While in healthy individuals this ecosystem maintains a stable symbiosis, disturbances in its composition—known as dysbiosis—can precipitate the onset of oral diseases such as caries and periodontal disorders. Gingivitis alone affects an estimated 3.5 billion people worldwide, making it one of the most widespread conditions across all populations and age groups, according to the World Health Organization (WHO) [1].

At the cellular level, gingivitis is characterized by the inflammatory response of the gingival tissue to biofilm accumulation. This process involves the release of pro-inflammatory cytokines (e.g., IL-1β, IL-6, TNF-α) and matrix metalloproteinases (MMPs), as well as increased oxidative stress, all contributing to tissue breakdown and further disease progression [2,3,4,5]. Microbiologically, a shift occurs from the predominance of Gram-positive commensal bacteria toward Gram-negative anaerobes, including well-documented periodontal pathogens such as *Porphyromonas gingivalis* and *Treponema denticola* [6,7]. Clinically, this transition manifests as gingival redness, swelling, bleeding during brushing, and, in some cases, gingival recession. If left untreated, gingivitis can evolve into periodontitis, leading to irreversible damage to the alveolar bone. The role of dental plaque in the pathogenesis of periodontal disease has been well established for decades [8].

Effective prevention begins with consistent oral hygiene practices. The WHO strongly advocates daily brushing, interdental cleaning, and regular dental visits as foundational strategies. However, beyond mechanical plaque removal, maintaining the balance of the oral microbiota is increasingly recognized as essential [9]. Conventional chemical adjuncts, such as chlorhexidine-containing mouthwashes, demonstrate clear efficacy but are associated with drawbacks—mucosal irritation, alterations in taste, dental staining [10,11], and disruption of the beneficial oral microbiota [12,13]. Given these limitations, attention has turned toward natural ingredients capable of maintaining antimicrobial efficacy without disturbing the oral microbiota. Over the past decade, several natural or herbal mouthwashes have been investigated as alternatives to chemical formulations, including those based on green tea, aloe vera, propolis, and essential oils. These preparations demonstrated variable efficacy in reducing plaque and gingival inflammation [14,15,16,17]. Among the wide variety of natural sources explored, marine organisms—in particular algae—offer a biochemical diversity shaped by harsh oceanic environments, making them a promising yet underutilized resource for oral care applications. Marine-derived bioactives thus represent a novel class of compounds with unique structural features and biological properties, potentially offering broad anti-inflammatory and microbiota-balancing effects.

Marine algae represent an underexplored yet promising source of bioactive compounds. Among these, phlorotannins are unique polyphenolic compounds exclusive to certain brown algae species, with *Fucus vesiculosus* being one of the richest natural sources [18]. Phlorotannins are three-dimensional polymers of phloroglucinol and exhibit strong anti-inflammatory properties [19,20]. Fucoidans, the main class of polysaccharides found in brown algae, are rich in L-fucose and sulfate ester groups and demonstrate a range of anti-inflammatory and immunomodulatory effects [21,22]. While preclinical studies have reported promising effects of these marine compounds against oral pathogens and inflammation [23,24,25,26], robust clinical evidence supporting their use in human oral health remains limited.

*Silybum marianum* is a flowering plant primarily found along oceanic coasts. Its main active components are phenolic compounds, most notably kaempferol, silymarin, and taxifolin, as well as polysaccharides. Silymarin is a complex mixture of flavonolignans composed of silibinin A, silibinin B, isosilibinin A, isosilibinin B, silydianin, and silychristin. Studies have demonstrated that *Silybum marianum* extracts possess antimicrobial activity [27,28], and exhibit antibiofilm properties [29]. Importantly, antibacterial effects targeting oral pathogens have also been reported [30,31], suggesting potential applications in oral health care.

Overall, while several herbal mouthwashes have demonstrated benefits in plaque and gingivitis control, the use of marine-derived bioactives for oral care remains largely unexplored. The present study thus addresses this gap by evaluating, for the first time in humans, a marine mouthwash formulation that combines *Fucus vesiculosus* and *Silybum marianum* extracts with seawater for dual anti-inflammatory and antimicrobial activity. The rationale for this combination lies in their complementary properties—anti-inflammatory activity from *Fucus vesiculosus* and seawater, and antibacterial effects from *Silybum marianum*—identified through preliminary in vitro screening conducted by our team. Key endpoints include gingival inflammation, plaque accumulation, halitosis, oral microbiota composition, and overall patient satisfaction. We hypothesize that this natural marine mouthwash will reduce gingival inflammation and plaque while exerting a milder impact on the oral microbiota compared with conventional chemical formulations.

## 2. Materials and Methods

### 2.1. Study Design

This randomized, parallel-group, open-label, single-center clinical trial was conducted from 2 July 2024 to 11 December 2024 at a private dental practice (Rennes, France). Three investigators were involved in conducting the study. To avoid inter-examiner variability, each participant was assessed and followed exclusively by a single investigator from baseline to the final visit. The study was conducted in accordance with Good Clinical Practice and in agreement with the Declaration of Helsinki. The study protocol was approved by the research ethics committee of the Catholic University of the West (Université Catholique de l’Ouest, Angers, France) under the code 2024-11/01 and was registered on clinicaltrials.gov (NCT06808711). Written informed consent was obtained from all subjects.

### 2.2. Eligibility Criteria

The inclusion and exclusion criteria were selected to keep the study population homogenous and minimize confounding factors that could influence the clinical effects of mouthwash. Participants were eligible for inclusion in the study if they met the following criteria: age between 18 and over, with sensitive gums, identified by a gingival index ≥2 (according to Loe & Silness scale) on at least 10% of teeth (i.e., 3 teeth). Written informed consent was obtained from all subjects. The exclusion criteria were as follows: individual who has regularly used a mouthwash during the past month, individual suffering from periodontitis, individual scheduled for a scaling procedure during the session, or who has undergone a scaling procedure within the 6 months preceding the baseline visit, individual who has received antibiotic or antifungal treatment within the 3 months preceding the visit, individual receiving anticoagulant, antiplatelet, immunosuppressive, or anticancer treatment (chemotherapy or radiotherapy of the head and neck), individual at risk of infective endocarditis, individual with known sensitivity or allergy to any of the mouthwashes components, volunteer participating in an interventional research study.

### 2.3. Intervention

Participants in the Test group used the marine mouthwash under investigation, which contains diluted seawater, glycerin, sodium benzoate/potassium sorbate, sodium fluoride, *Fucus vesiculosus* extract rich in polysaccharides (alginate, laminaran et fucoidans), *Silybum marianum* extract, Tween 20, and mint aroma. The *Fucus vesiculosus* and *Silybum marianum* extracts were selected following in vitro screening of several natural candidates for two complementary activities relevant to gingivitis management: anti-inflammatory and antibacterial. The selected *Fucus vesiculosus* extract demonstrated strong anti-inflammatory effects on human gingival cells, while the *Silybum marianum* extract exhibited significant antibacterial activity against *Porphyromonas gingivalis*. These extracts showed the best efficacy-to-concentration ratio among tested candidates.

Participants in the Comparator group used a mouthwash available on the international market. It contains sorbitol, propylene glycol, sodium lauryl sulfate, Poloxamer 407, benzoic acid, sodium fluoride, eucalyptol, zinc chloride, methyl salicylate, thymol, sodium saccharin, sodium benzoate, menthol, aroma, benzyl alcohol, sucralose and green coloring agents. This mouthwash contains essential oils (eucalyptol, thymol, methyl salicylate, menthol), which provide antiseptic properties, and sodium fluoride, which provides anticaries action. Benzyl alcohol is usually present as a preservative.

### 2.4. Randomization

The participants were randomly assigned to one of the two groups. The investigators gave the corresponding mouthwash to each patient.

Randomization was performed without stratification by sex or age, as these variables were not expected to influence short-term gingival or plaque outcomes.

The allocation sequence was generated using a computer-generated random number sequence, and assignments were implemented using pre-prepared randomization list.

### 2.5. Study Protocol

**Figure 1:**
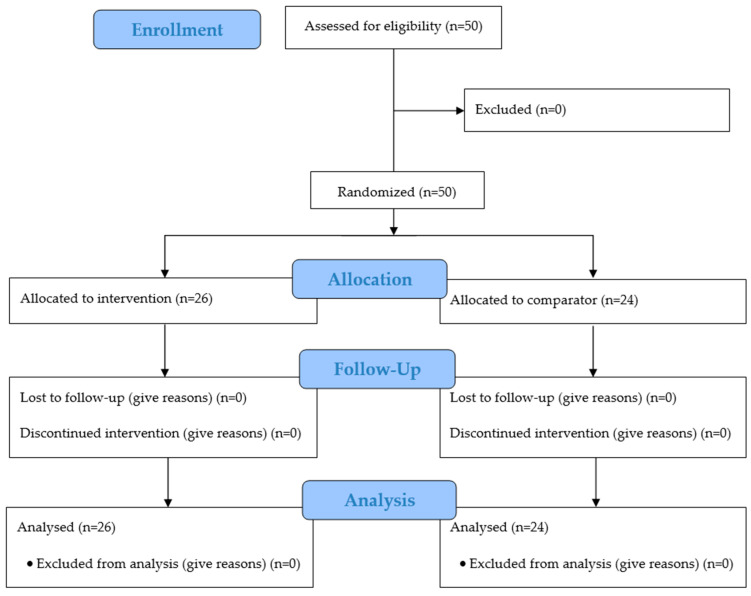
CONSORT flow diagram showing the study planning.

The subjects for this study were recruited from among patients of the outpatient clinic in a single center. Patients who met the inclusion criteria were enrolled in this study (Figure 1). At the first visit (T0), the clinical evaluation was carried out, and two swab samples were collected from each participant for microbiota analysis (participants did not brush their teeth immediately prior to sampling). Sampling was standardized and performed by dry swabbing using sterile swabs. For each sample, the swab was passed over the occlusal surfaces, followed by a firm pass over the vestibular surfaces at the gingival margin on teeth 17 to 27 and then 37 to 47. The procedure was repeated with a second swab to obtain the second sample.

Subsequently, the marine mouthwash and the comparator mouthwash were distributed to the patients according to the allocation scheme. All participants were asked to brush their teeth with the toothpaste provided by the investigator and instructed to use the mouthwash twice daily after toothbrushing (morning and evening), with a volume of 15 mL for 7 days (T7). The mouthwashes were to be used undiluted for 30 s and then spat out. After 7 days, the participants were recalled and questioned for any inconvenient incidents during the study period. A clinical examination and swab sampling were conducted.

No formal sample size calculation was performed prior to the study; the number of participants was determined based on feasibility and availability during the recruitment period. This is acknowledged as a limitation of the study.

### 2.6. Clinical Evaluation

The clinical evaluations were performed at baseline (T0) and after 7 days (T7).

- Gingival index (GI) by Loe and Silness [32] was measured on each tooth on the mesio-buccal surfaces (one site per tooth) using a probe. The scores were given as follows: 0—normal gingiva, no inflammation; 1—mild inflammation, no bleeding; 2—moderate inflammation, erythema, bleeding on probing; 3—severe inflammation, severe erythema and swelling, tendency to spontaneous bleeding.
- Plaque index (PI) by Silness and Loe [33] was measured on each tooth on the mesio-buccal surfaces (one site per tooth). The following scores were given: 0—no plaque; 1—a thin layer of plaque only detected by scraping with a probe; 2—moderate accumulation of plaque within gingival pocket, plaque is visible to the naked eye; 3—plaque presence around the gingival margin with vast majority of interdental spaces filled with plaque.
- Halitosis (bad breath) was measured using the spoon test originating from the posterior part of the tongue dorsum [34]. The patients were asked to refrain from drinking, eating, chewing gum, rinsing the mouth, gargling, and smoking for at least 2 h before performing the test. A clean plastic spoon was used to scrape the back of the tongue. After about 5 s, the odor of the spoon’s contents was evaluated by a third party (a close contact of the volunteer participating in the study), holding the spoon approximately 5 cm from the nose. The third party assessed the intensity of the breath using the following organoleptic scale: 0—no odor; 1—questionable odor; 2—slight malodor; 3—moderate malodor; 4—strong malodor; 5—severe malodor.

### 2.7. Microbiological Analysis

Total DNA from the swab samples was extracted using the DNeasy^®^ Blood & Tissue Kit (Qiagen, Courtaboeuf, France) and PCR-amplified with PuReTaq^TM^ Ready-To-Go^TM^ PCR beads (Cytiva, Saint-Germain-en-Laye, France) following manufacturer protocols. The V1–V3 regions of the 16S rRNA gene were amplified with primers 8F (5′-AGA-GTT-TGA-TCC-TGG-CTC-AG-3′) and 534R (5′-ATT-ACC-GCG-GCT-GCT-GG-3′) with 25 cycles of PCR at 60 °C. Positive (ZymoBIOMICS DNA Standard, Zymo Research, Freiburg, Germany) and negative controls were included. Amplicons were sequenced on the Illumina MiSeq at the EcogenO facility (University of Rennes, Biogenouest Genomics, AnaEE-Fr, Rennes, France).

FASTQ files were processed in QIIME2 (v. 2023.9) [35] as ‘PairedEndFastqManifestPhred33’. Sequence quality was filtered with DADA2 [36], truncating reads at 285 bp (forward) and 271 bp (reverse). Sample counts ranged from 1444 and 59,149. Data are available in the NCBI SRA (BioProject PRJNA1309332).

Core diversity analyses included alpha diversity (Faith’s PD, Pielou’s evenness) [37,38], beta diversity (Bray–Curtis, weighted/unweighted UniFrac) [39,40]. Alpha diversity was compared with Wilcoxon rank-sum tests (FDR-corrected) [41]. Beta diversity was assessed with PERMANOVA and PERMDISP in the QIIME2 diversity plugin. PCoA plots were generated with Bray–Curtis and UniFrac distances.

For taxonomy, reference sequences from eHOMD (V15.22.p9) matching primers 8F/534R were used to train as a Naïve Bayes classifier (V15.22 taxonomy). Feature Tables were exported at genus and species levels for further analysis. Microbial abundances were explored in R studio (version 2024.04.2+764).

### 2.8. Statistical Analysis

Statistical analysis was carried out using JMP® software, version 18.1.0 (SAS Institute, Cary, NC, USA) and R software (version 4.3.2; R Core Team 2023). Any p-values of less than 0.05 (*p* < 0.05) were considered statistically significant.

- For comparisons of independent data (intergroup comparisons): comparison of means will be performed using the Student t-test, after checking the assumptions. If assumptions are not met, the non-parametric Wilcoxon Mann–Whitney test will be used. Percentages will be compared using the Chi-square test after verifying the assumptions. If not met, Fisher’s exact test will be used.
- For paired comparisons (intragroup comparisons): percentages will be compared using McNemar test or McNemar–Bowker test.

## 3. Results

### 3.1. Demographic Characteristics at T0

In the Test group, the male/female ratio was 53.8%/46.2%. In the Comparator group, the ratio was 33.3%/66.7%.

The mean age in the Test group was 35.7 ± 19.7 years, with a median of 28.0 years (18–85). The mean age in the Comparator group was 41.7 ± 16.5 years, with a median of 39.0 years (18–77).

Regarding smoking status, 76.9% of volunteers included in the Test groups were non-smokers and 83.3% in the Comparator group.

None of the patients had diabetes or obesity.

### 3.2. Global Oral Health Status at T0

The number of teeth was very similar between the two groups (Test group: 27.5 ± 4.0 teeth and Comparator group: 27.3 ± 2.3 teeth, see Table 1), with an identical median of 28 teeth.

The number of volunteers with untreated caries was low, totaling 5 volunteers (i.e., 10% of all volunteers).

### 3.3. Gingival Index

#### 3.3.1. Gingival Index for All Teeth

In the Test group, 86.6% of teeth had a gingival index of grade 0 or 1 on Day 7 compared to 70.5% on Day 0 (see Table 2). In the Comparator group, 81.6% of teeth had a gingival index of grade 0 or 1 on Day 7 compared to 64.3% on Day 0.

Conversely, for grades 2 and 3, which are the most unfavorable, the number of affected teeth decreased between Day 0 and Day 7. In the Test group, the percentage of teeth with a gingival index of grades 2 and 3 decreased from 29.5% on Day 0 to 13.4% on Day 7, representing a 55% reduction. In the Comparator group, the percentage of teeth with a gingival index of grades 2 and 3 was reduced by 49% between Day 0 and Day 7 (35.7% on Day 0 vs. 18.3% on Day 7).

On Day 7, the distribution of grades was statistically different between the two groups, in favor of the Test group (*p* = 0.0238).

#### 3.3.2. Results for Teeth with Gingivitis

A focus on teeth with a gingival index of 2 or 3 at inclusion was conducted. For each patient, the mean score of these teeth was calculated on Day 0 and Day 7 by multiplying each score (2 or 3) by the number of associated teeth, then dividing the total by the number of teeth with a gingival index of 2 or 3. Mean of these scores was calculated for all patients on Day 0 and Day 7.

**Figure 2:**
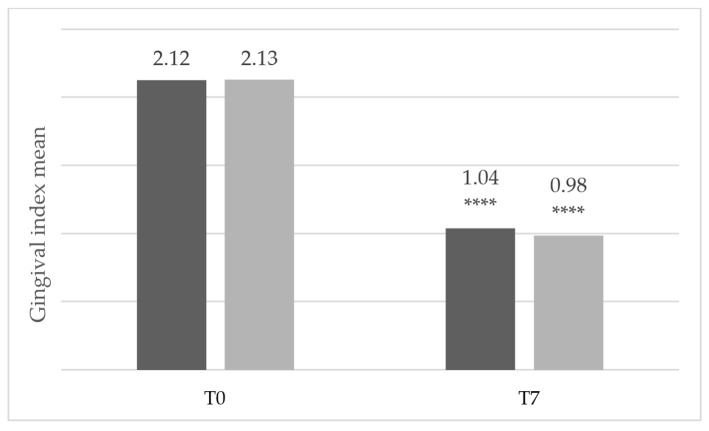
Mean gingival index scores of teeth with a gingival index of 2 or 3 at inclusion (dark gray: Test group, *n* = 26; light gray: comparator group, *n* = 24), **** *p* < 0.0001 between Day 0 and Day 7. Exact *p*-values: Test group = 1.19 × 10^−7^; Comparator group = 2.38 × 10^−7^.

Figure 2 shows that the mean score of teeth with a gingival index of 2 or 3 at inclusion (all patients as it was an inclusion criterion) decreased from 2.12 to 1.04 in the Test group and from 2.13 to 0.98 in the Comparator group. No statistically significant difference was observed between the two groups on Day 7.

### 3.4. Plaque Index

#### 3.4.1. Plaque Index for All Teeth

In the Test group, 81.7% of teeth had a plaque index of grade 0 or 1 on Day 7 compared to 71.4% on Day 0 (see Table 3). In the Comparator group, 79.1% of teeth had a plaque index of grade 0 or 1 on Day 7 compared to 66.6% on Day 0.

Conversely, for grades 2 and 3, which are the most unfavorable, the number of affected teeth decreased between Day 0 and Day 7. In the Test group, the percentage of teeth with a plaque index of grades 2 and 3 decreased from 28.6% on Day 0 to 18.3% on Day 7, representing a 36% reduction. In the Comparator group, the percentage of teeth with a gingival index of grades 2 and 3 was reduced by 37% between Day 0 and Day 7 (33.4% on Day 0 vs. 20.9% on Day 7).

On Day 7, the distribution of grades was statistically different between the two groups, in favor of the Test group (*p* = 0.0017).

#### 3.4.2. Results for Teeth with Dental Plaque

A focus on teeth with a plaque index of 2 or 3 at inclusion was conducted. For each patient, the mean score of these teeth was calculated on Day 0 and Day 7 by multiplying each score (2 or 3) by the number of associated teeth, then dividing the total by the number of teeth with a plaque index of 2 or 3. Mean of these scores was calculated for all patients on Day 0 and Day 7.

**Figure 3:**
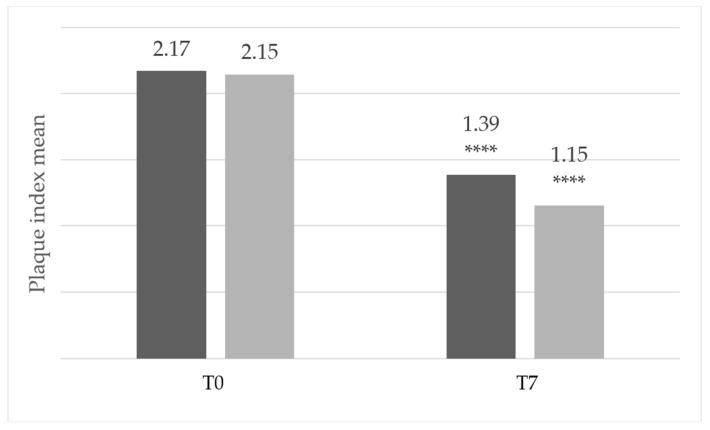
Mean plaque index scores of teeth with a plaque index of 2 or 3 at inclusion (dark gray: Test group, *n* = 24; light gray: comparator group, *n* = 23). The number of patients with dental plaque was 47, which is lower than the total study population because the presence of dental plaque was not an inclusion criterion. **** *p* < 0.0001 between Day 0 and Day 7. Exact *p*-values: Test group = 6.10 × 10^−5^; Comparator group = 1.91 × 10^−6^.

Figure 3 shows that the mean score of teeth with a plaque index of 2 or 3 at inclusion decreased from 2.17 to 1.39 in the Test group and from 2.15 to 1.15 in the Comparator group. No statistically significant difference was observed between the two groups on Day 7.

### 3.5. Halitosis

The results of the spoon test for halitosis assessment are presented in Table 4. At inclusion, the self-assessment results of breath were similar between the two groups. Most participants did not report halitosis (score 0 and 1) at inclusion: 53.8% in the Test group and 50% in the Comparator group.

In the Test group, 73.1% of participants reported no odor or a questionable odor on Day 7 compared to 53.8% on Day 0. In the Comparator group, 87.5% of participants reported no odor or a questionable odor on Day 7 compared to 50.0% on Day 0. No statistically significant difference was observed between Day 0 and Day 7, nor between the two groups.

### 3.6. Safety

No adverse effects were reported by the participants in the Test group.

In the comparator group, one adverse effect was reported by one participant. It was mouth ulcers after 3 days.

### 3.7. Microbiota

#### 3.7.1. Alpha Diversity Analyses

**Figure 4:**
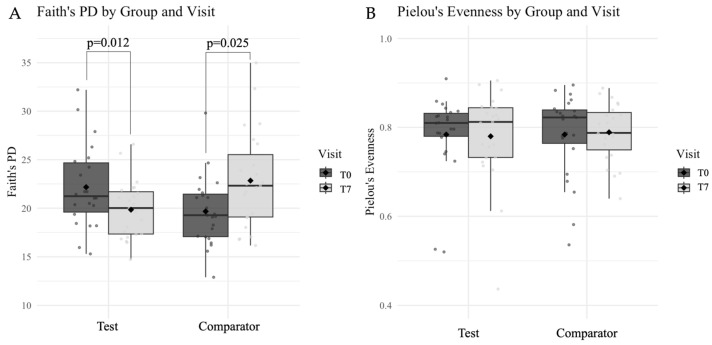
Box-plots with median representing the (**A**) richness and (**B**) evenness of the taxa present in each group (Test *n* = 23; Comparator *n* = 23) and visit (dark gray: T0; light gray: T7). The black point on each box-plot indicates the calculated mean of the respective group.

At inclusion (T0), there was no difference in microbial richness measured between Test and Comparator groups (*p* = 0.053, Table 5 and Figure 4A). The Test marine mouthwash induced a significant decrease (*p* = 0.012, Table 5 and Figure 4A) in phylogenetic diversity. In contrast, the microbial richness significantly increased with the Comparator mouthwash (*p* = 0.025, Table 5 and Figure 4A) from baseline. With regard to microbial evenness, no difference was found between groups before or after treatment (Table 5 and Figure 4B).

#### 3.7.2. Beta Diversity Analyses

**Figure 5:**
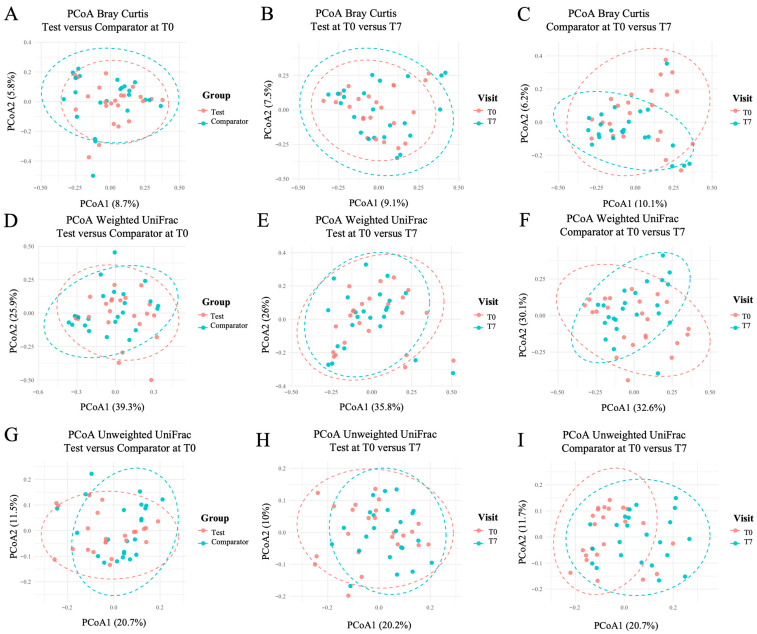
Principal Coordinate Analysis (PCoA) plots of Bray–Curtis (**A**–**C**), weighted UniFrac (**D**–**F**) and unweighted UniFrac (**G**–**I**). Comparisons: Test versus Comparator at T0 (**A**,**D**,**G**); Test T0 versus T7 (**B**,**E**,**H**); and Comparator T0 versus T7 (**C**,**F**,**I**). Ellipses indicate group means with 95% confidence intervals.

There was no difference in microbial composition at inclusion (T0) between Test and Comparator groups (*p* > 0.05, Table 6). *Beta* diversity analyses showed a significant shift in microbial composition in the Comparator group between T0 and T7 according to PERMANOVA for Bray–Curtis (*p* = 0.021, Table 6), Weighted UniFrac (*p* = 0.033, Table 6) and Unweighted UniFrac (*p* = 0.001, Table 6). Dispersion tests (PERMDISP; *p* > 0.05, Table 6) confirmed that these differences were not due to heterogeneity within groups. No significant differences in microbial composition were observed in the Test group over time. Figure 5 presents the Principal Coordinate Plots (PCoA) of the different *beta* diversity results and the comparison groups of interest.

#### 3.7.3. Microbial Taxa and Differential Abundance Exploration

**Figure 6:**
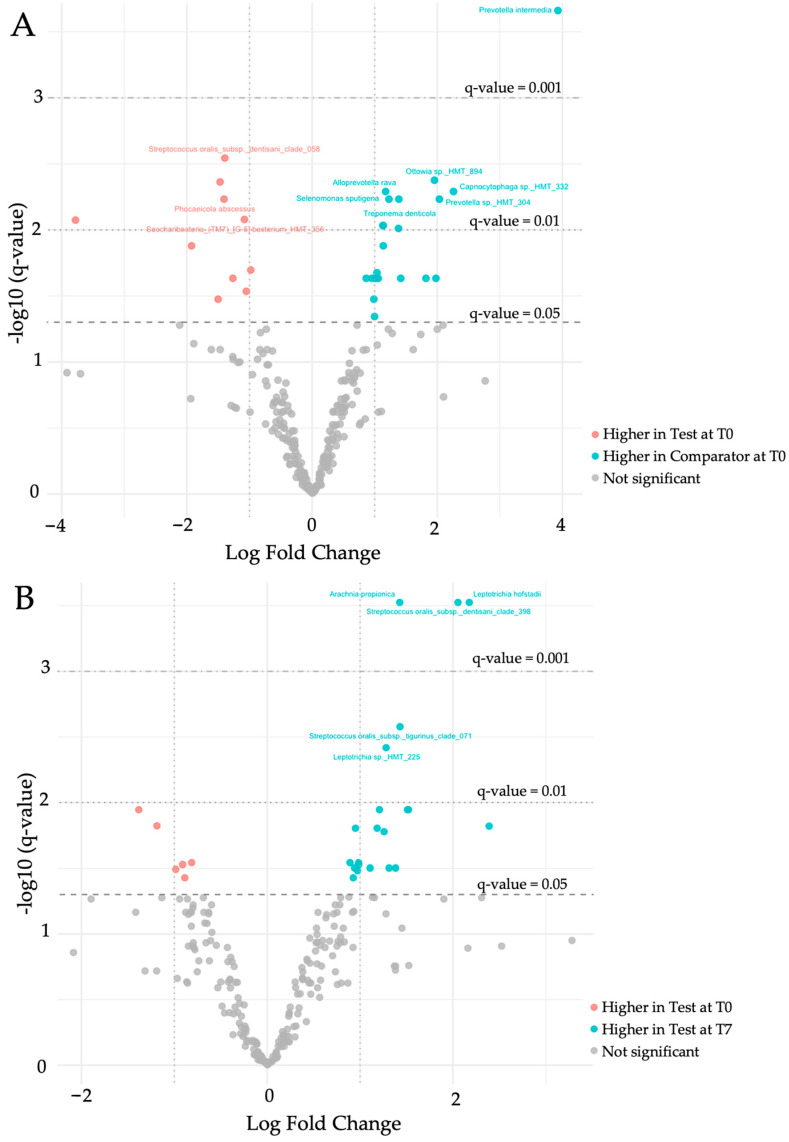

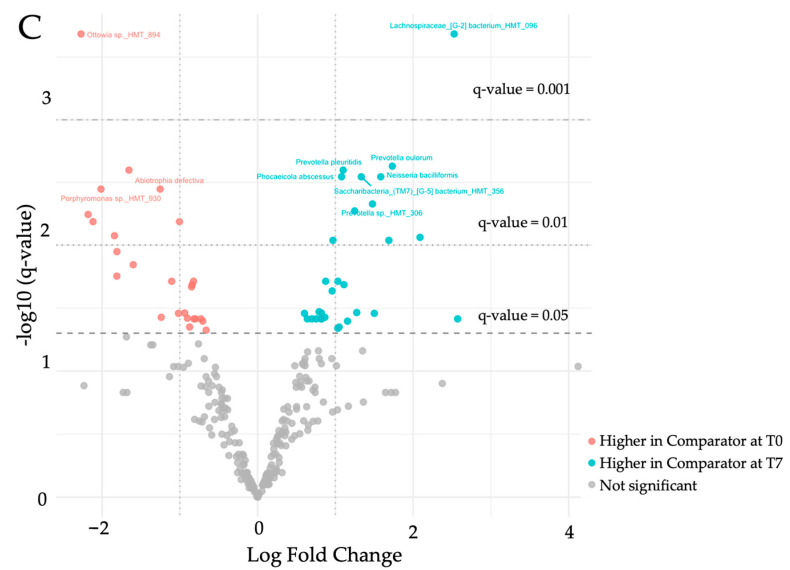
Volcano plots of differentially abundant taxa between (**A**) Test versus Comparator at T0, (**B**) Test T0 versus T7, and (**C**) Comparator T0 versus T7. The *x*-axis shows log_2_ fold change in relative abundance, and the *y*-axis shows -log_10_ FDR-adjusted q-values. Dotted lines mark significance thresholds (q = 0.05, 0.01, 0.001). Taxa above q < 0.05 are considered significant; the top 10 with q < 0.01 are labeled. Higher-level taxa (Genus, Family, Order, etc.) were excluded from labels.

To analyze the microbial composition between groups across visits, differential abundance was processed by Analysis of Composition of Microbiomes with bias correction (ANCOM-BC) [42]. Comparisons were made between:

(i) Test vs. Comparator at inclusion (Figure 6A, Supplementary Table S1A),
(ii) Test T0 vs. T7 (Figure 6B, Supplementary Table S1B), and
(iii) Comparator T0 vs. T7 (Figure 6C, Supplementary Table S1C).

At inclusion, disease-associated taxa (*Prevotella intermedia*, *Treponema denticola*, *Alloprevotella rava*) were more abundant in the Comparator group, while more health-associated taxa (*Streptococcus oralis subsp. dentisani* and *Granulitella adiacens*) were more represented in the Test group.

After treatment with the Test marine mouthwash, additional health-associated and commensal taxa became significantly enriched (Supplementary Table S1B). In contrast, the Comparator mouthwash led to a complete shift in bacterial species, affecting both healthy- and disease-associated taxa, without changing the overall ratio of health- to disease-associated taxa (Supplementary Table S1C). Notably, *Prevotella* and *Treponema* species as well as *Fusobacterium nucleatum subsp. animalis* increased in the Comparator group.

#### 3.7.4. Effect of Mouthwash Treatments on *Porphyromonas* Species

*Porphyromonas* species were selected as a biomarker of periodontal health.

**Figure 7:**
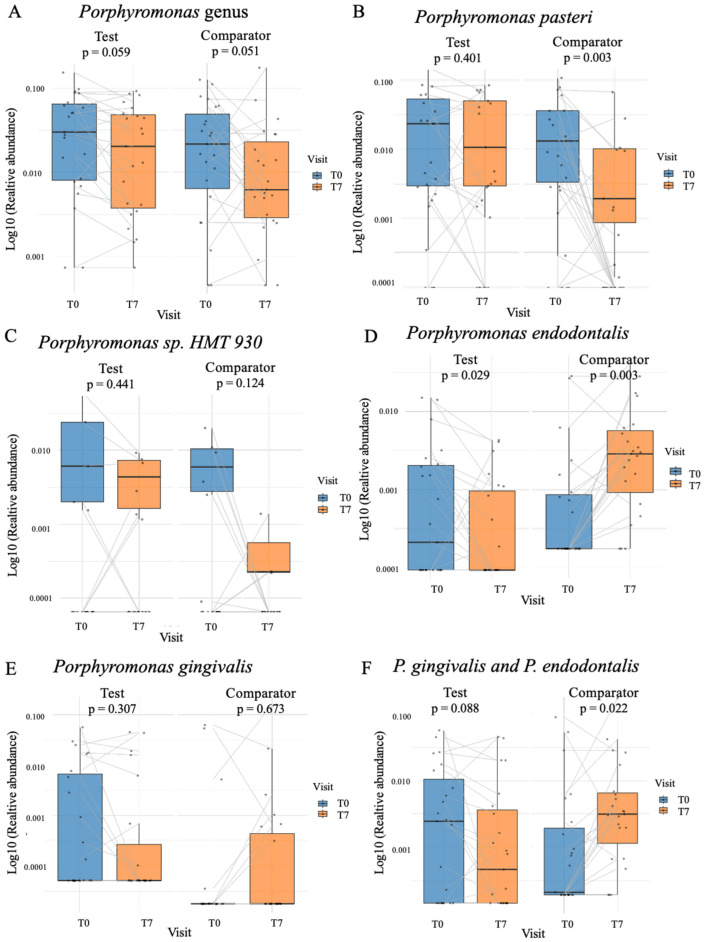
Changes in relative abundance of *Porphyromonas* taxa following mouthwash use. Box-plots representing relative abundances of (**A**) *Porphyromonas* genus, (**B**) *P. pasteri*, (**C**) *Porphyromonas* sp. HMT 930, (**D**) *P. endodontalis*, (**E**) *P. gingivalis* and (**F**) combined *P. gingivalis* and *P. endodontalis*, at baseline (T0; blue boxes) and after 7 days (T7; orange boxes) of intervention. Individual lines connect paired samples of the same participant over time. Comparisons were performed separately for the Test and Comparator groups using paired statistical tests; *p*-values are presented above each panel.

At the genus level, both groups showed a trend toward decreased abundance (Figure 7A; Test: *p* = 0.059 and Comparator: *p* = 0.051). At the species level, *P. pasteri* significantly decreased in the Comparator group (Figure 7B; *p* = 0.003), while *Porphyromonas* sp. HMT 930 showed no significant changes in either group (Figure 7C).

Interestingly, *P. endodontalis* significantly decreased in the Test group (Figure 7D; *p* = 0.029) while increased in Comparator groups (Figure 7D; *p* = 0.003). *P. gingivalis* abundance remained non-significant, but similar patterns were observed in favor of the marine mouthwash (Figure 7E).

To account for the low baseline detection of the *Porphyromonas* periopathogen (ei.: *P. gingivalis* and *P. endodontalis)* a combined analysis was performed. This combined abundance was significantly increased in the Comparator group (Figure 7F; *p* = 0.022) but decreased in the marine mouthwash group (Figure 7F; *p* = 0.088).

Table 7 summarizes the proportion of participants showing reduced or increased *Porphyromonas* abundances. A majority of participants tested with the Test marine mouthwash (73%) showed a decrease in *P. gingivalis*, while 71% of participants in the Comparator group showed an increase (*p* = 0.1448). In contrast, 80% of Test participants showed a mean decrease of −44% ± 66% in *P. endodontalis*, versus an 85% increase in the Comparator group (*p* = 0.0002). The combined analysis of *P. gingivalis* and *P. endodontalis* also demonstrated a significant reduction in the Test group compared with the Comparator (*p* = 0.0009).

## 4. Discussion

This randomized clinical trial evaluated the efficacy and safety of a novel natural ingredients-based mouthwash containing a marine algae extract, a coastal plant extract, and seawater compared to a conventional comparator mouthwash in subjects with moderate to severe gingivitis. The results demonstrate a significant improvement in gingival index and plaque index scores in both groups after 7 days of use, with a more pronounced reduction in higher gingival index grades (2 and 3) observed with the marine mouthwash. These findings suggest that the marine formulation exhibits effective anti-inflammatory and anti-plaque properties, which align with preclinical evidence supporting the benefits of brown algae-derived polysaccharides and phlorotannins. To further address gingival inflammation and microbial dysbiosis, the formulation was designed to combine two bioactives with distinct and complementary mechanisms—anti-inflammatory and antimicrobial—although synergistic effects were not formally assessed.

The microbial findings are consistent with the clinical outcomes: participants using the marine mouthwash exhibited both reduced plaque and gingival indices, along with a decrease in disease-associated taxa such as *P. endodontalis*. These complementary results suggest that the clinical improvements observed are partly mediated by the re-establishment of a healthier microbial balance, highlighting the functional link between oral microbiota modulation and gingival inflammation reduction.

The plausible mechanism of action involves bioactive constituents such as fucoidans and phlorotannins from *Fucus vesiculosus*, which have demonstrated anti-inflammatory effects through modulation of pro-inflammatory cytokines and immunomodulatory activity [19,20,21,22,23,24,25,26]. The selective antimicrobial properties that may preserve oral microbiota balance are attributed to the *Silybum marianum* extract [27,28,29,30,31]. This may explain the favorable tolerability and absence of adverse events with the marine mouthwash, contrasting with conventional mouthwashes which often cause mucosal irritation, taste alteration, or staining [43,44].

Although reductions in halitosis prevalence were noted in both groups, these changes did not reach statistical significance. However, the overall improvement in gingival health and plaque control likely contributes to better oral health outcomes and indirectly impacts halitosis, consistent with known pathophysiological links between gingival inflammation and oral malodor [45,46].

Although microbial richness tended to differ between groups at baseline (T0), the evolution of microbial diversity metrics was completely opposite in the Test and Comparator groups after 7 days of mouthwash use. The marine mouthwash was associated with reduced microbial richness and stability of community structure, while the Comparator increased richness and induced compositional shifts. Differential abundance analyses suggested species-specific effects: *P. endodontalis* consistently decreased with the marine mouthwash, and *P. gingivalis* showed a trend toward reduction, with a higher proportion of decreases in the Test group compared with the Comparator group. When combined, these disease-associated taxa were significantly reduced with the marine mouthwash, despite high inter-individual variability. By contrast, *P. pasteri*, a health-associated species, was reduced only in the Comparator group. However, these microbiota changes observed over a short 7-day period should be considered preliminary. Most of the key clinical outcomes and several microbiota comparisons yielded very low *p*-values (often <0.001), which would remain significant even under conservative multiple-testing corrections. Therefore, we consider it appropriate to describe these differences as statistically significant, while acknowledging that microbiota analyses were exploratory and should be interpreted with caution. Longer-term studies are required to determine whether such compositional shifts translate into sustained oral health benefits and clinically meaningful outcomes.

A study by Laumen et al. (2024) reported that the use of an alcohol-containing mouthwash significantly altered the oral microbiome, with an increased abundance of *Fusobacterium nucleatum* and *Streptococcus anginosus* [47]. The authors suggested that alcohol-related intake/use may increase the abundance of cancer-associated bacteria. In our study, the Comparator mouthwash that contains alcohol appeared to alter drastically the microbiome stucture, giving rise to a number of disease-associated species, including *F. nucleatum subsp. animalis*. This particular sub-species of *F. nucleatum* was recently described to be implicated in human colorectal cancer development due to its ability to proliferate during inflammation [48,49,50]. Beyond alcohol, other ingredients commonly found in mouthwashes have also raised concerns. A previous report has also raised concerns that daily use of mouthwashes, particularly those containing chlorhexidine or essential oils, may promote oral dysbiosis [51]. Similarly, it has been demonstrated that sodium lauryl sulfate disturbed the microbial community structure by increasing the number of pathogenic bacteria [52]. In contrast, the Test marine mouthwash was associated with an enrichment of health-associated and commensal species, suggesting it may contribute to the maintenance and/or establishment of a more balanced, less dysbiotic microbiome.

Our findings are in line with previous research on natural mouthwashes [14,15,16]. More recently, Lile et al. (2025) conducted a randomized clinical trial comparing herbal mouthwashes with chlorhexidine and reported reductions in dental plaque and gingival inflammation, while highlighting improved tolerability of natural formulations [17]. These results, together with our study, support growing evidence that plant-based and marine-derived mouthwashes can provide effective alternatives to conventional chemical products, offering clinical benefits with few adverse effects.

The study limitations include the short duration (7 days), which restricts conclusions on long-term gingival health maintenance and periodontal disease progression prevention. Additionally, the modest sample size may limit the statistical power to detect differences in parameters such as halitosis, which could be assessed in the future using objective instrumental measurements. Neither participants nor evaluators were blinded to group allocation, which may introduce some risk of performance or assessment bias. However, the primary outcomes (plaque and gingival indices) were based on objective clinical measurements using standardized indices, which reduces the likelihood of significant bias.

Future studies with larger cohorts and extended follow-up durations are warranted to confirm these promising findings and further elucidate the interactions between marine bioactives, oral microbiota, and host immune responses.

## 5. Conclusions

The marine mouthwash, formulated with a marine algae extract, a coastal plant extract, and seawater demonstrated clinically meaningful reductions in gingival inflammation and dental plaque after one week of use, with an excellent safety and tolerability profile. These results suggest that natural, algae-derived oral care products may represent a promising alternative or adjunct to conventional chemical mouthwashes for short-term management of gingivitis.

Beyond its clinical efficacy, the marine mouthwash showed promising and hypothesis-generating effects on oral microbiota composition, favoring the enrichment of health-associated bacterial taxa and reducing disease-associated species such as *Porphyromonas endodontalis*. This microbiological profile suggests a potential role in restoring oral microbial balance and preventing dysbiosis-related complications.

Taken together, these results support the integration of algae-based formulations into daily oral hygiene routines to promote the periodontal health and enhance patient quality of life.

Further research should focus on long-term clinical outcomes and microbiological assessments to substantiate these initial findings and expand clinical applications.

## Figures and Tables

**Table 1:** Description of the oral health status at T0.

Population		Test Group (*n* = 26)	Comparator Group (*n* = 24)
Number of teeth	Mean	27.5	27.3
	Standard deviation	4.0	2.3
	Median	28.0	28.0
Number of teeth with untreated cavities	0 cavity	22 (84.6%)	23 (95.8%)
	1 cavity	3 (11.5%)	1 (4.2%)
	4 cavities	1 (3.8%)	0

**Table 2:** Gingival index for all teeth in both groups.

% of Teeth	Timeline	Test Group (*n* = 26)	Comparator Group (*n* = 24)
Gingival index of 0 and 1	T0	70.5	64.3
	T7	86.6	81.6
Gingival index of 2 and 3	T0	29.5	35.7
	T7	13.4	18.3
	*p*-value between groups	0.0238	

**Table 3:** Plaque index for all teeth in both groups.

% of Teeth	Timeline	Test Group (*n* = 26)	Comparator Group (*n* = 24)
Plaque index of 0 and 1	T0	71.4	66.6
	T7	81.7	79.1
Plaque index of 2 and 3	T0	28.6	33.4
	T7	18.3	20.9
	*p*-value between groups	0.0017	

**Table 4:** Breath evaluation in both groups.

	Test Group (*n* = 26)		Comparator Group (*n* = 24)	
	T0	T7	T0	T7
0—no odor	7 (26.9%)	6 (23.1%)	4 (16.7%)	5 (20.8%)
1—questionable odor	7 (26.9%)	13 (50.0%)	8 (33.3%)	16 (66.7%)
2—slight malodor	5 (19.2%)	6 (23.1%)	6 (25.0%)	2 (8.3%)
3—moderate malodor	6 (23.1%)	1 (3.8%)	4 (16.7%)	0
4—strong malodor	1 (3.8%)	0	2 (8.3%)	1 (4.2%)
5—severe malodor	0	0	0	0

**Table 5:** Calculated means ± SD of alpha-diversity metrics measuring richness (Faith’s pd) and evenness (Pielou’s) of the taxa present in each group. (* *p* ≤ 0.05 = significant).

Diversity Metric	Group	T0	T7	*p*-Value
Faith’s pd (richness)	Test	22.12 ± 4.21	19.85 ± 2.99	0.012 *
	Comparator	19.67 ± 3.55	22.84 ± 5.04	0.025 *
	***p*-value**	0.053	-	-
Pielou’s Evenness	Test	0.78 ± 0.10	0.78 ± 0.10	0.988
	Comparator	0.78 ± 0.09	0.79 ± 0.07	0.988
	***p*-value**	0.617	-	-

**Table 6:** Summary of *beta*-diversity tests (PERMANOVA, PERMDISP; 999 permutations) using Bray–Curtis, weighted and unweighted UniFrac. Pairwise comparisons were performed within each. Significant differences (*p* ≤ 0.05) are marked with an asterisk (*).

		PERMANOVA			PERMDISP	
Diversity Metric	Comparison	Pseudo-F Statistic	R^2^	*p* Value	F-Statistic	*p* Value
Bray Curtis	Test T0 vs. Comparator T0	0.955	0.021	0.552	0.007	0.938
	Test T0 vs. T7	0.485	0.011	1.000	0.779	0.391
	Comparator T0 vs. T7	1.477	0.032	0.021 *	0.172	0.674
Weighted UniFrac	Test T0 vs. Comparator T0	0.758	0.017	0.554	0.105	0.749
	Test T0 vs. T7	0.301	0.007	0.960	0.011	0.908
	Comparator T0 vs. T7	2.655	0.057	0.033 *	0.109	0.726
Unweighted UniFrac	Test T0 vs. Comparator T0	1.324	0.029	0.153	0.468	0.493
	Test T0 vs. T7	1.250	0.028	0.184	1.759	0.182
	Comparator T0 vs. T7	3.110	0.066	0.001 *	2.389	0.116

**Table 7:** Presence and changes in *P. gingivalis* only, *P. endodontalis* only, or both *P. gingivalis* and *P. endodontalis* following mouthwash use. This Table summarizes overall detection, presence at baseline (T0), and day 7 (T7), and the proportion of participants showing increased or reduced relative abundance at T7 in the Test and Comparator groups. Fisher’s exact test (*) comparing increased versus reduced relative abundances between Test and Comparator groups, # Wilcoxon Rank Sum Test. The mean percentage reduction ± SD in relative abundance is reported.

Taxa	Treatment	Presence Overall	Presence at T0	Presence at T7	Increased at T7 *	Reduced at T7*	*p*-Value *	Change in Relative Abundance #
*P. gingivalis*	Test	11/23 (47%)	11/11 (100%)	7/11 (63%)	3/11 (27%)	8/11 (73%)	0.1448	−33% ± 72%
	Comparator	7/23 (30%)	4/7 (57%)	7/7 (100%)	5/7 (71%)	2/7 (29%)		+46% ± 88%
*P. endodontalis*	Test	15/23 (65%)	12/15 (80%)	15/15 (100%)	3/15 (20%)	12/15 (80%)	0.0002	−44% ± 66%
	Comparator	20/23 (87%)	9/20 (45%)	20/20 (100%)	17/20 (85%)	3/20 (15%)		+63% ± 53%
*P. gingivalis and* *P. endodontalis*	Test	17/23 (73.9%)	15/17 (88%)	14/17 (82%)	4/17 (23.5%)	13/17 (76.5%)	0.0009	−32% ± 69%
	Comparator	20/23 (86.9%)	12/20 (60%)	20/20 (100%)	16/20 (80%)	4/20 (20%)		+60% ± 65%

## Data Availability

Data is contained within the article, in Supplementary Material and on the NCBI SRA server (BioProject PRJNA1309332).

## Supplementary Materials

The following supporting information can be downloaded at: https://www.mdpi.com/article/10.3390/dj14010002/s1, Table S1: Differentially abundant taxa by ANCOM-BC analysis.

## Abbreviations

The following abbreviations are used in this manuscript:

WHO	World Health Organization
MMP	Matrix Metalloproteinases
GI	Gingival index
PI	Plaque index
PCR	Polymerase Chain Reaction
