# Supplementary material for: Control of Dental Plaque and Gingival Inflammation by Natural Ingredients-Based Mouthwash
Source: Dent J (Basel). 2025 Dec 19;14(1):2. doi: 10.3390/dj14010002 (PMC12839718; doi:10.3390/dj14010002)
Supplement: Supplementary file 1 [file dentistry-14-00002-s001.zip › dentistry-3922769-supplementary.pdf]

Supplementary Table S1 : Differentially abundant taxa by ANCOM-BC analysis.

(A) Test versus Comparator at T0			
**Taxon**	**q-value**	**Higher in**	**Association**
Streptococcus oralis subsp. dentisani clade 058	0.003	Test	Health
Parvimonas (Genus)	0.004	Test	Disease
Phocaeicola abscessus	0.006	Test	Commensal / Unknown
Saccharibacteria (TM7) [G-5] bacterium HMT 356	0.008	Test	Disease
Lachnoanaerobaculum saburreum	0.008	Test	Disease
Capnocytophaga sp. HMT 412	0.013	Test	Commensal / Unknown
Saccharibacteria (TM7) [G-1] bacterium HMT 348	0.020	Test	Commensal / Unknown
Treponema sp. HMT 262	0.023	Test	Disease
Leptotrichia wadei	0.029	Test	Disease
Veillonellaceae [G-1] bacterium HMT 150	0.033	Test	Disease
Prevotella intermedia	0.0002	Comparator	Disease
Ottowia sp. HMT 894	0.004	Comparator	Health
Alloprevotella rava	0.005	Comparator	Disease
Capnocytophaga sp. HMT 332	0.005	Comparator	Health
Treponema denticola	0.006	Comparator	Disease
Selenomonas sputigena	0.006	Comparator	Disease
Prevotella sp. HMT 304	0.006	Comparator	Disease
Pseudoleptotrichia sp. HMT 219	0.009	Comparator	Health
Peptostreptococcaceae [G-9] [Eubacterium] brachy	0.009	Comparator	Disease
Prevotella micans	0.010	Comparator	Disease
Veillonella sp. HMT 780	0.013	Comparator	Disease
Fretibacterium fastidiosum	0.021	Comparator	Disease
Desulfobulbus sp. HMT 041	0.023	Comparator	Disease
Haemophilus sputorum	0.023	Comparator	Health
Actinomyces sp. HMT 897	0.023	Comparator	Health
Alloprevotella tannerae	0.023	Comparator	Disease
Capnocytophaga sputigena	0.023	Comparator	Disease
Peptostreptococcaceae [G-7] bacterium HMT 081	0.023	Comparator	Disease
Peptostreptococcaceae [G-6] [Eubacterium] nodatum	0.023	Comparator	Disease
Mogibacterium timidum	0.033	Comparator	Disease
Desulfovibrio (Genus)	0.045	Comparator	Disease

(B) Test at T0 versus T7			
**Taxon**	**q-value**	**Higher in**	**Association**
Clostridiales [F-1][G-1] bacterium HMT 093	0.011	Test T0	Disease
Porphyromonas sp. HMT 930	0.015	Test T0	Disease
Peptostreptococcaceae [G-7] [Eubacterium] yurii subsps. yurii & margaretiae	0.029	Test T0	Disease
Absconditabacteria (SR1) [G-1] bacterium HMT 874	0.030	Test T0	Disease
Alloprevotella (Genus)	0.032	Test T0	Disease
Prevotella micans	0.037	Test T0	Commensal / Unknown
Streptococcus oralis subsp. dentisani clade 398	0.0003	Test T7	Health
Arachnia propionica	0.0003	Test T7	Commensal / Unknown
Leptotrichia hofstadii	0.0003	Test T7	Commensal / Unknown
Streptococcus oralis subsp. tigurinus clade 071	0.003	Test T7	Disease
Leptotrichia sp. HMT 225	0.004	Test T7	Health
Desulfobulbus sp. HMT 041	0.011	Test T7	Disease
Saccharibacteria (TM7) [G-1] bacterium HMT 347	0.011	Test T7	Disease
Stomatobaculum sp. HMT 097	0.011	Test T7	Health
Cardiobacterium valvarum	0.015	Test T7	Disease
Prevotella maculosa	0.016	Test T7	Health
Peptostreptococcaceae [G-7] (Genus)	0.016	Test T7	Disease
Prevotella koreensis	0.017	Test T7	Commensal / Unknown
Corynebacterium durum	0.029	Test T7	Health
Prevotella jejuni	0.029	Test T7	Commensal / Unknown
Desulfovibrio (Genus)	0.031	Test T7	Disease
Streptococcus oralis subsp. tigurinus clade 070	0.031	Test T7	Health
Streptococcus infantis clade 638	0.031	Test T7	Health
Capnocytophaga sp. HMT 412	0.031	Test T7	Commensal / Unknown
Alloprevotella sp. HMT 914	0.033	Test T7	Disease
Capnocytophaga endodontalis	0.037	Test T7	Disease

(C) Comparator at T0 versus T7			
**Taxon**	**q-value**	**Higher in**	**Association**
Ottowia sp. HMT 894	0.0002	Comparator T0	Commensal / Unknown
Alloprevotella (Genus)	0.003	Comparator T0	Disease
Abiotrophia defectiva	0.004	Comparator T0	Disease
Porphyromonas sp. HMT 930	0.004	Comparator T0	Commensal / Unknown
Veillonella sp. HMT 780	0.006	Comparator T0	Commensal / Unknown
Ruminococcaceae [G-1] bacterium HMT 075	0.007	Comparator T0	Commensal / Unknown
Capnocytophaga sp. HMT 332	0.007	Comparator T0	Commensal / Unknown
Prevotella sp. HMT 304	0.008	Comparator T0	Disease
Selenomonas dianae	0.011	Comparator T0	Disease
Peptostreptococcaceae [G-7] bacterium HMT 081	0.014	Comparator T0	Disease
Prevotella micans	0.018	Comparator T0	Disease
Schaalia sp. HMT 180	0.019	Comparator T0	Health
Porphyromonas (Genus)	0.019	Comparator T0	Disease
Peptostreptococcaceae [G-4] (Genus)	0.021	Comparator T0	Disease
Streptococcus parasanguinis clade 721	0.021	Comparator T0	Disease
Granulicatella elegans	0.035	Comparator T0	Health
Streptococcus oralis subsp. tigurinus clade 070	0.035	Comparator T0	Health
Haemophilus haemolyticus	0.037	Comparator T0	Health
Selenomonas sputigena	0.038	Comparator T0	Disease
Haemophilus (Genus)	0.039	Comparator T0	Health
Bacteroidetes [G-5] bacterium HMT 511	0.039	Comparator T0	Disease
Prevotella saccharolytica	0.039	Comparator T0	Disease
Selenomonas noxia	0.045	Comparator T0	Disease
Mogibacterium timidum	0.047	Comparator T0	Disease
Lachnospiraceae [G-2] bacterium HMT 096	0.0002	Comparator T7	Disease
Prevotella oulorum	0.002	Comparator T7	Commensal / Unknown
Prevotella pleuritidis	0.003	Comparator T7	Disease
Neisseria bacilliformis	0.003	Comparator T7	Disease
Phocaeicola abscessus	0.003	Comparator T7	Commensal / Unknown
Saccharibacteria (TM7) [G-5] bacterium HMT 356	0.003	Comparator T7	Disease
Prevotella sp. HMT 306	0.005	Comparator T7	Disease
Neisseria sp. HMT 018	0.009	Comparator T7	Commensal / Unknown
Actinomyces johnsonii	0.009	Comparator T7	Health
Streptococcus sp. HMT 057	0.009	Comparator T7	Commensal / Unknown
Lachnoanaerobaculum orale	0.019	Comparator T7	Disease
Bacteroidetes [G-5] bacterium HMT 505	0.019	Comparator T7	Disease
Prevotella jejuni	0.021	Comparator T7	Commensal / Unknown

Capnocytophaga gingivalis	0.023	Comparator T7	Disease
Saccharibacteria (TM7) [G-6] bacterium HMT 870	0.034	Comparator T7	Disease
Prevotella oralis	0.034	Comparator T7	Disease
Parvimonas (Genus)	0.035	Comparator T7	Disease
Pseudoramibacter alactolyticus	0.035	Comparator T7	Disease
Fusobacterium nucleatum subsp. animalis	0.035	Comparator T7	Disease
Prevotella sp. HMT 472	0.037	Comparator T7	Disease
Megasphaera micronuciformis	0.039	Comparator T7	Health
Arachnia rubra	0.039	Comparator T7	Commensal / Unknown
Capnocytophaga sp. HMT 412	0.039	Comparator T7	Commensal / Unknown
Capnocytophaga sp. HMT 336	0.039	Comparator T7	Health
Prevotella sp. HMT 443	0.039	Comparator T7	Disease
Lancefieldella parvula	0.039	Comparator T7	Commensal / Unknown
Treponema sp. HMT 262	0.040	Comparator T7	Disease
Streptococcus sanguinis	0.045	Comparator T7	Health
Veillonellaceae [G-1] (Genus)	0.046	Comparator T7	Disease
